# Exhaled 8-isoprostane in childhood asthma

**DOI:** 10.1186/1465-9921-6-79

**Published:** 2005-07-21

**Authors:** Sukhbir K Shahid, Sergei A Kharitonov, Nicola M Wilson, Andrew Bush, Peter J Barnes

**Affiliations:** 1 Section of Airway Disease, National Heart and Lung Institute, Imperial College London, London, UK; 2 Department of Paediatrics, Royal Brompton Hospital, London, UK

**Keywords:** oxidative stress, 8-isoprostane, exhaled breath condensate, childhood asthma

## Abstract

**Background:**

Exhaled breath condensate (EBC) is a non-invasive method to assess airway inflammation and oxidative stress and may be useful in the assessment of childhood asthma.

**Methods:**

Exhaled 8-isoprostane, a stable marker of oxidative stress, was measured in EBC, in children (5–17 years) with asthma (13 steroid-naïve and 12 inhaled steroid-treated) and 11 healthy control.

**Results:**

Mean exhaled 8-isoprostane concentration was significantly elevated in steroid-naïve asthmatic children compared to healthy children 9.3 (SEM 1.7) vs. 3.8 (0.6) pg/ml, p < 0.01. Children on inhaled steroids also had significantly higher 8-isoprostane levels than those of normal subjects 6.7 (0.7) vs. 3.8 (0.6) pg/ml, p < 0.01. Steroid-naïve asthmatics had higher exhaled nitric oxide (eNO) than those of controls 28.5 (4.7) vs. 12.6 (1.5) ppb, p < 0.01. eNO in steroid-treated asthmatics was similar to control subjects 27.5(8.8) vs. 12.6(1.5) ppb. Exhaled 8-isoprostane did not correlate with duration of asthma, dose of inhaled steroids or eNO.

**Conclusion:**

We conclude that 8-isoprostane is elevated in asthmatic children, indicating increased oxidative stress, and that this does not appear to be normalized by inhaled steroid therapy. This suggests that 8-isoprostane is a useful non-invasive measurement of oxidative stress in children and that antioxidant therapy may be useful in the future.

## Introduction

Anti-inflammatory drugs such as inhaled corticosteroids now are the mainstay of treatment in childhood asthma but measurement of airway inflammation using traditional invasive procedures is not feasible in children. Bronchial biopsy is invasive and non-invasive tests such as spirometry do not represent the true state of inflammation [1]. Less invasive tests such as sputum induction may be difficult in children.

Exhaled breath condensate analysis is simple to perform, is effort-independent, non-invasive and rapid [2,3]. Various mediators of inflammation and oxidative stress, such as hydrogen peroxide, cysteinyl-leukotrienes, 8-isoprostanes have been measured in exhaled breath condensate and have been found to be elevated in adults with asthma compared to values in normal control subjects [4,5]. However, there are few studies of exhaled breath condensate in children.

Increased oxidative stress is a feature of airway inflammation in asthma and inflammatory cells, such as eosinophils, neutrophils, macrophages, and mast cells all produce reactive oxygen radicals [6]. 8-isoprostane is a stable product formed by oxidative metabolism of arachidonic acid and appears to be a reliable marker of oxidative stress [7]. 8-isoprostane is increased in exhaled breath condensate in adult asthmatic and its concentration is related to asthma severity [4]. We therefore measured 8-isoprostane in exhaled breath of children with asthma who were either steroid-naïve or treated with inhaled steroids. We compared exhaled 8-isoprostane with the levels of nitric oxide (NO) in exhaled air as this has previously been used as a non-invasive marker of airway inflammation in asthma.

## Methods

### Study population

Normal and asthmatic children 2–18 years of age who were able to co-operate with the measurements were enrolled into the study. The diagnosis of asthma was based on a history of repetitive cough, breathlessness and wheeze responsive to bronchodilators with or without inhaled steroid [8]. Children with concomitant chronic airway disease like cystic fibrosis or those suffering from an exacerbation during the study period were excluded. Stable steroid-treated asthmatic children were recruited from the Pediatric Outpatient Clinics of Royal Brompton Hospital. The asthmatic children not on inhaled steroids (steroid-naïve) and normal controls were recruited from a local church community or from relatives of staff. Informed and written consent was obtained from parent/s or guardian/s and the study was approved by the Research Ethics Committee of Royal Brompton and Harefield NHS Trust.

### Study design

Children on inhaled corticosteroids (n = 12) were divided into two groups; those on ≤ 600 μg/day (low-dose group, n = 6) and those on >600 μg/day (high-dose group, n = 6). Atopic status was assessed by skin prick test to 4 common allergens (grass pollen, house dust mite, cat hair and *Aspergillus fumigatus *[Alk Abello, Denmark]). Exhaled NO [9] and spirometry were measured; followed by collection of exhaled breath condensate. In a separate group of 10 steroid-treated asthmatic children, the exhaled breath condensate collections were performed twice 10 minutes apart to assess the reproducibility of the test.

### Exhaled NO measurement

Exhaled NO was measured by a single breath technique using a chemiluminescence analyser (NiOx analyzer, Aerocrine, Stockholm, Sweden) at an expired flow of 50 ml/second. This equipment has a sensitivity of ± 1.5 ppb and a precision of ± 2.5 ppb. A direct digital reading is obtained and average of two readings was taken in each child. As spirometric maneuvers are known to affect NO readings, exhaled NO measurement was done prior to lung function testing.

### Spirometry

The airway function determination was performed by dry spirometer (Vitalograph, Buckingham, UK). The highest of three consecutive measurements was taken. FEV_1 _% predicted was calculated for each child.

### Exhaled breath condensate analysis

Subjects breathed tidally for 10 minutes into the condenser (Ecoscreen, Jaeger, Hoechberg, Germany), wearing a nose clip. Exhaled condensate was frozen at -20°C elsius, after defrosting the sample was aliquoted into small plastic tubes and stored at -80°C for later analysis. This has been shown not to affect 8-isoprostane concentrations over six months of storage [10].

### 8-isoprostane assay

8-isoprostane was assayed by an enzyme-linked immunosorbent assay (Cayman Chemical, Ann Arbor, MI). The lowest detection limit of the assay was 4.5 pg/ml and the intra and interassay correlation coefficient was ≤ 10% [10,11].

### Data analysis

All data are expressed as means ± SEM. Comparison of demographic data was done by Chi-square test. Continuous data of two subgroups were tested for significant difference by unpaired Student's t-test for normally distributed data. Correlations were performed by Pearson's test for normally distributed data, and by Spearman's test for non-Gaussian data. Significance was considered when p < 0.05. Reproducibility was assessed by Bland-Altman plot of the paired values of exhaled 8-isoprostane of the asthmatic children [10,11].

## Results

We studied 11 normal control subjects, 13 steroid-naïve and 12 steroid-treated asthmatic children. There was no significant difference in age and sex of children between these three groups (Table 1). There were 11 mild intermittent and 2 moderate persistent asthmatics in the steroid-naïve group. In the steroid-treated group, 6 children were using ≤ 600 μg/day of inhaled corticosteroids, and 6 were using >600 μg/day. All the children in steroid-treated asthmatic group were well-controlled. 24 of the subjects were skin prick test positive to at least one of the 4 common tested aeroallergens (grass pollen, house dust mite, cat hair and *Aspergillus fumigatus)*. The mean eNO was significantly raised in steroid-naïve asthmatic children (28.5 ± 4.7 ppb) compared to normal subjects (12.6 ± 1.5 ppb, p < 0.01), but was not significantly increased in the steroid-treated group compared to the healthy controls (27.5 ± 8.8 ppb, p = 0.11). There was a wide scatter of eNO levels in the steroid-treated group.

**Table 1:** Clinical characteristics of study population

Parameter	Normal Controls	Steroid-naïve asthmatic children	Steroid-treated asthmatic children	p value
Number	11	13	12	-
Females	7	8	7	NS
Mean age (years)	11.4	10.8	10.7	NS
Atopic children	5	12	7	NS
Mean FEV1 (% Predicted)	85	83	90	NS
Mean exhaled NO (ppb)	11.9	28.5*	27.5	<0.01

*p < 0.05 compared to levels in normal children.

NS = non-significant.

**Figure 1:**
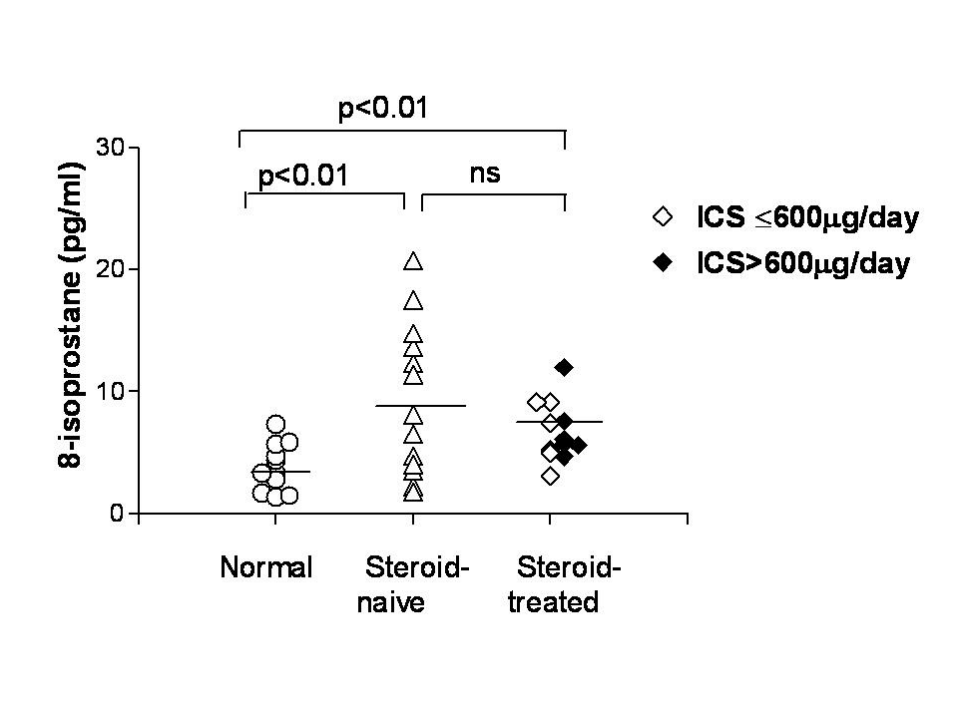
Exhaled 8-isoprostane in normal, steroid-naïve asthmatic and steroid-treated asthmatic children.

8-isoprostane was detected in 24/36 subjects. Mean exhaled 8-isoprostane was higher in asthmatic children who were not on inhaled steroid therapy compared to that in the normal children (9.3 ± 1.7 vs 3.8 ± 0.6 pg/ml, p < 0.01). The asthmatic children using inhaled steroids had a trend towards lower 8-isoprostane levels than that of steroid-naïve group (6.7 ± 0.7 vs 9.3 ± 1.7 pg/ml respectively, p = 0.18); their results were significantly higher than in control subjects (6.7 ± 0.7 vs 3.8 ± 0.6 pg/ml, p < 0.01) (Fig. 1).

**Figure 2:**
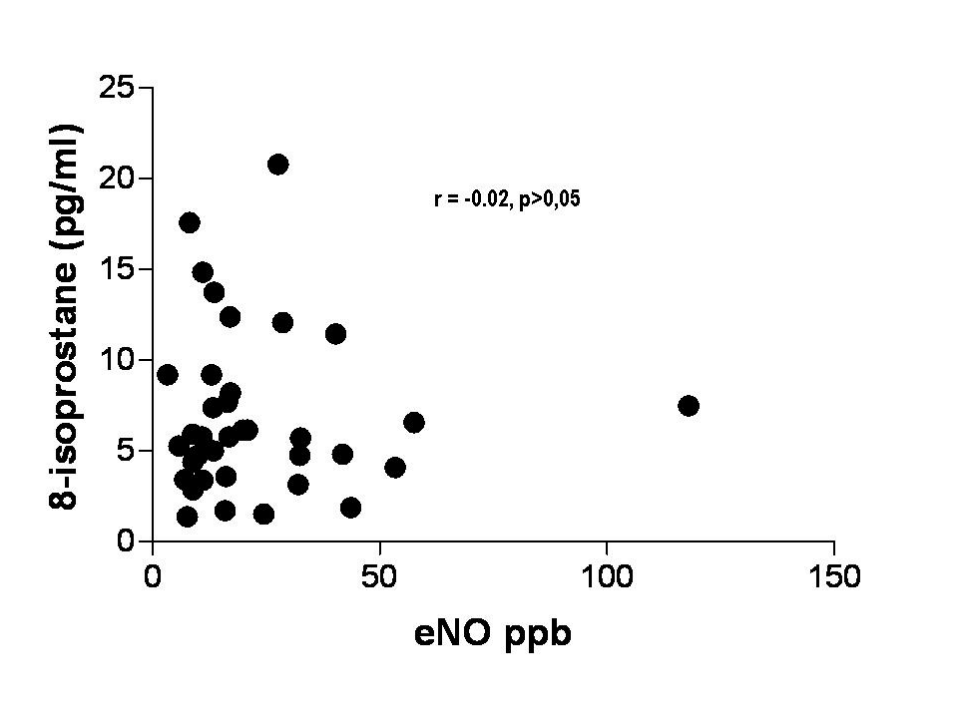
Correlation between exhaled nitric oxide (NO) and exhaled 8-isoprostane in children (r = -0.02, p = non-significant).

There was no correlation between 8-isoprostane concentrations and exhaled NO (Fig. 2) or FEV_1_% predicted. 8-isoprostane did not correlate with duration of asthma or the prescribed dose of inhaled steroids. The levels of exhaled 8-isoprostane in the low-dose and high-dose subgroups of steroid-treated asthmatic children were 6.5 ± 1.0 and 7.0 ± 1.1 pg/ml respectively (p > 0.05).

The second group of asthmatic children on whom reproducibility of the measurements was analyzed comprised of 10 inhaled steroid-treated asthmatics (4 females, mean age 12.3 years, range 10 to 14.5 years). The doses of inhaled steroids in these children ranged from 200 to 2000 μg/day. We found that the Bland-Altman plot of repeat values of 8-isoprostane performed 10 minutes apart in these 10 asthmatic children demonstrated good reproducibility. The intra-class correlation coefficient (ICC) of the two readings was 0.98 and the correlation coefficient (r) was 0.98 (95% confidence interval of 0.92 to 1.00).

## Discussion

We have shown that 8-isoprostane is detectable in exhaled breath condensate of children, with significantly higher concentrations in exhaled breath of asthmatic children compared to healthy controls. Steroid-treated asthmatic patients had a trend towards lower levels of 8-isoprostane in exhaled breath, although the concentrations were still significantly higher than in normal subjects. When the exhaled breath condensate was assessed for reproducibility of 8-isoprostane it was found to be highly reproducible (ICC = 0.98).

We studied 36 children, including steroid-naïve asthmatics, asthmatic patients on inhaled steroids and normal controls. The children were age and sex-matched and the youngest child who was able to undertake the test was 5 years. Measurement of 8-isoprostane is a useful marker to assess the oxidative stress of asthma *in vivo*, since it is a stable product of oxidative metabolism. Our study revealed that asthmatic children had significantly higher levels of exhaled 8-isoprostane than those in healthy volunteers. 8-isoprostane is predominantly formed by oxidative metabolism of arachidonic acid via a non-enzymatic reaction, but a small amount of 8-isoprostane may be formed by a cyclooxygenase pathway [7]. Measurement of 8-isoprostane appears to be a reliable biomarker of oxidative stress. The higher concentrations of exhaled 8-isoprostane in exhaled breath condensates of children with asthma indicates increased oxidative stress in asthmatic airways. The levels are lower but still significantly greater than normal in children treated with inhaled steroid therapy, indicating that anti-inflammatory treatment does not abolish oxidative stress even their asthma control appears good. However measurement of 8-isoprostane in a group of asthmatic children before and after the initiation of steroids is needed in future studies to determine the response to steroid therapy. In the present study, there was no correlation between exhaled 8-isoprostane and the dose of inhaled steroids in steroid-treated asthmatic children. However, we were unable to compare 8-isoprostane concentrations with those in the bronchoalveolar lavage fluid, as the latter is difficult to perform and not ethically justifiable purely for research in children. In our study, no correlation was seen between 8-isoprostane and exhaled NO or FEV_1_% predicted. This temporal measurement would enable us to determine the utility of exhaled 8-isoprostane in predicting an attack of asthma. In the steroid-naïve group most patients were mild and we did not have enough patients with severe disease to study whether exhaled 8-isoprostane levels reflects disease severity as in adults [4]. We found that exhaled breath condensate measurement of 8-isoprostane was highly reproducible when repeated measurements were made in 10 asthmatic subjects.

We found that eNO was significantly higher in asthmatic children compared to normal controls, but were reduced in steroid-treated patients, in agreement with other studies [12]. Other authors have measured active oxygen radicals and documented oxidative stress in asthma [13]. We have previously reported increased concentrations of 8-isoprostane in adult asthmatics, but the exhaled 8-isoprostane concentrations were higher compared to those in our study. This could be due to the different age groups studied and the fact that the adult patients had more severe asthma. The raised levels of exhaled 8-isoprostane despite inhaled steroid therapy is consistent with the supposition that 8-isoprostane is derived predominantly via the non-enzymatic peroxidation. This elevation in exhaled 8-isoprostane in steroid-treated asthmatics is consistent with the studies of other authors [14,15]. In our study, it can be seen that eNO and 8-isoprostane appear to respond differently to inhaled steroid therapy and no correlation existed between eNO and exhaled 8-isoprostane. This indicates that a single mediator cannot serve as a global marker of inflammation in asthma and it may be necessary to measure more than one exhaled marker.

Our study shows that elevated 8-isoprostane is detectable in the exhaled breath condensates of children with asthma and may be used as a non-invasive measurement of oxidative stress in childhood asthma. The persistence of high levels of 8-isoprostane in spite of inhaled steroids suggests that other treatments such as anti-oxidants might be beneficial.
